# Self-Reported Modifiable Risk Factors of Cardiovascular Disease among Older Adults in Malaysia: A Cross-Sectional Study of Prevalence and Clustering

**DOI:** 10.3390/ijerph18157941

**Published:** 2021-07-27

**Authors:** Ying Ying Chan, Norhafizah Sahril, Muhammad Solihin Rezali, Lim Kuang Kuay, Azli Baharudin, Mohamad Aznuddin Abd Razak, Mohd Shaiful Azlan Kassim, Muhammad Fadhli Mohd Yusoff, Mohd Azahadi Omar, Noor Ani Ahmad

**Affiliations:** 1 Centre for Family Health Research, Institute for Public Health, National Institutes of Health, Ministry of Health Malaysia, Setia Alam, Shah Alam 40170, Malaysia; norhafizah_s@moh.gov.my (N.S.); muhammadsolihin@moh.gov.my (M.S.R.); aznuddin.ar@moh.gov.my (M.A.A.R.); shaiful.azlan@moh.gov.my (M.S.A.K.); 2 Centre for Occupational Health Research, Institute for Public Health, National Institutes of Health, Ministry of Health Malaysia, Setia Alam, Shah Alam 40170, Malaysia; limkk@moh.gov.my; 3 Centre for Nutrition Epidemiology Research, Institute for Public Health, National Institutes of Health, Ministry of Health Malaysia, Setia Alam, Shah Alam 40170, Malaysia; ps_azlibaharudin@moh.gov.my; 4 Centre for Non-Communicable Diseases Research, Institute for Public Health, National Institutes of Health, Ministry of Health Malaysia, Setia Alam, Shah Alam 40170, Malaysia; fadhli_my@moh.gov.my; 5 Sector for Biostatistics & Data Repository, National Institutes of Health, Ministry of Health Malaysia, Setia Alam, Shah Alam 40170, Malaysia; drazahadi@moh.gov.my; 6 Director Office, Institute for Public Health, National Institutes of Health, Ministry of Health Malaysia, Setia Alam, Shah Alam 40170, Malaysia; drnoorani@moh.gov.my

**Keywords:** cardiovascular disease, modifiable risk factors, prevalence, older adults, National Health and Morbidity Survey, Malaysia

## Abstract

The co-occurrence of multiple modifiable risk factors increases the risk of cardiovascular disease (CVD) morbidity or mortality. This study examines the prevalence and clustering of self-reported modifiable CVD risk factors among older adults in Malaysia. A total of 7117 adults aged ≥50 years participated in the National Health and Morbidity Survey (NHMS) 2018: Elderly Health, a community-based cross-sectional survey. Data were collected using a standardized structured questionnaire. Multivariable logistic regression was used to determine the factors associated with the clustering of self-reported modifiable CVD risk factors. The prevalence of self-reported diabetes, hypertension, hypercholesterolemia, overweight/obesity, and current smoking was 23.3%, 42.2%, 35.6%, 58.4%, and 17.5%, respectively. Overall, the prevalence of clustering of ≥1, ≥2, and ≥3 modifiable CVD risk factors was 83.3%, 75.4%, and 62.6%, respectively. Multivariable logistic regression analysis showed that men, 60–69 age group, urban dwellers, having no formal education, unemployed/retirees/homemakers, and being physically inactive were independently associated with self-reported modifiable CVD risk factors clustering. There are also ethnic differences in self-reported modifiable CVD risk factors clustering. Our findings underscore the necessity of targeted interventions and integrated strategies for early detection and treatment of modifiable CVD risk factors among older adults, considering age, sex, ethnicity, and socioeconomic status.

## 1. Introduction

Cardiovascular disease (CVD) remains the leading global cause of death in both developed and developing countries, with an estimated 17.9 million deaths accounting for 32% of global mortality in 2019 [1]. The figure is predicted to increase to approximately 23 million deaths by 2030 [2]. Although recent studies suggest that all forms of CVDs have declined in developed countries [3,4], the prevalence is increasing in developing countries [5,6]. More than 80% of CVD-related deaths occur in low- and middle-income countries (LMICs) [7]. The burden of CVD is expected to immensely increase along with the growth of the aging population, and the increasing practice of unhealthy lifestyles, due to rapid industrialization and urbanization in LMICs. The age-related increase in CVD imposes a substantial burden on global health in terms of mortality, morbidity, disability, reduced functioning, and health care costs [8].

Malaysia, a multi-ethnic country with a growing aging population, is a middle-income country experiencing an increased prevalence of CVD risk factors, such as diabetes, hypertension, hypercholesterolemia, overweight/obesity, and smoking [9,10,11,12]. CVD has been the leading cause of morbidity and mortality in Malaysia for more than a decade [9,10,11,12]. Based on the 2019 Health Facts by the Ministry of Health Malaysia, mortality due to CVDs ranked first among the ten principal causes of death in government hospitals (21.65%) and ranked second in private hospitals (23.79%) [13]. With the rise in the aging population and increasing burden of CVD in Malaysia, the Malaysian government is expected to experience a greater healthcare expenditure to provide more inclusive healthcare for its population.

It is well known that major chronic diseases that burden the aging population in Malaysia were diabetes, hypertension, and hypercholesterolemia [9,10,11,12]. The five major CVD risk factors—diabetes, hypertension, hypercholesterolemia, overweight/obesity, and smoking—have been reported to show a clustering phenomenon that will increase the risk for CVD compared to a single risk factor [14]. Lifestyle factors, such as physical inactivity, low intake of fruits and vegetables, and excessive alcohol consumption are established modifiable risk factors for CVD [15]. A cohort study in the Asia-Pacific region suggested that all combinations of risk factors are associated with an increased risk of CVD, and that the risk increases in the presence of additional risk factors, with the clusters, including high blood pressure, associated with the highest additional risk of CVD [16]. Hence, knowledge and information on the burden of the clustering of CVD risk factors are important to help in devising appropriate and integrated healthcare planning for the prevention and management of CVD.

The Ministry of Health Malaysia has been regularly monitoring the prevalence of chronic diseases, patterns, and costs of healthcare utilization in the Malaysian population through the National Health and Morbidity Surveys (NHMSs). However, studies on the clustering of modifiable CVD risk factors in the Malaysian population are scarce. Three cross-sectional studies have reported the prevalence and clustering of CVD risk factors among the general adult population in Malaysia [14,17,18], and a longitudinal study by Thangiah et al. has reported the clustering of biological CVD risk factors and its’ transitions over time, among adolescents in Malaysia [19]. Another study by Majid et al. reported the prevalence and factors associated with self-reported diabetes, hypertension, and hypercholesterolemia among older persons aged ≥60 years in Malaysia [20]. There has been no study focusing on the clustering of modifiable CVD risk factors among older adults. Malaysia will be considered an aging nation by 2030, where 15% of its total population would be senior citizens aged 60 years and above [21]. Therefore, this study aims to determine the prevalence and clustering of self-reported modifiable CVD risk factors among adults aged 50 years and older in Malaysia. It was hypothesized that there will be a high prevalence of modifiable CVD risk factor clustering among older adults; and lower socioeconomic status, unhealthy diet, and inactive lifestyle will be associated with a greater number of CVD risk factors. Understanding how the clustering of modifiable CVD risk factors is associated with sociodemographic and lifestyle variables could help develop future interventions and prevention strategies to reduce the prevalence of CVDs.

## 2. Materials and Methods

### 2.1. Study Design and Participants

Data were obtained from the National Health and Morbidity Survey (NHMS) 2018, a nationwide cross-sectional study of community-dwelling adults aged 50 years and older. This survey employed a two-stage stratified random sampling method to ensure the national representativeness of the population aged ≥50 years. The primary stratum was made up of all states and Federal Territories, and the secondary stratum was made up of both urban and rural areas within the primary stratum. The survey used the National Household Sampling Frame made up of Enumeration Blocks (EB) containing 80 to 120 living quarters (LQ), each derived from the Population and Housing Census 2010, provided by the Department of Statistics Malaysia (DOSM). Samples were drawn independently within each level of the secondary stratum. The first stage of units of sample selection was the EB level, while the second stage units were the LQ within the EB. A total of 5636 eligible LQ were selected from 60 EB in urban areas and 50 EB in rural areas. All households and persons aged 50 years and above within the identified eligible LQ were included in the study, excluding those residing in institutions, such as hotels, hostels, hospitals, prisons, boarding houses, and nursing homes. The detailed methodology of the NHMS 2018 was described previously [22].

From July 2018 to September 2018, a total of 7427 individuals were eligible to participate in the survey; 7117 participants were successfully interviewed, giving an overall response rate of 95.8% (7117/7427). Written consents were obtained from all respondents prior to data collection. Illiterate respondents provided verbal consent with their thumbprint on the consent forms, and this is witnessed by a literate person. The study protocol was approved by the Medical Research and Ethics Committee (MREC), Ministry of Health Malaysia (NMRR-17-2655-39047).

### 2.2. Data Collection and Measurement

Data was collected via a face-to-face interview by trained interviewers using a standardized bilingual (Malay language and English) structured questionnaire. The questionnaire was programmed into an application called Survey Creation System (SCS), and the data collection was done using mobile devices, with real-time data entry to our server at the institute. For respondents with communication problems, such as post-stroke, cognitive impairment, or speech disabilities, or where there was a language barrier, their proxies who knew the respondents best were interviewed.

The structured questionnaire collected information on sociodemographic characteristics (age, sex, ethnicity, residential area, marital status, education level, employment status, individual monthly income, and living arrangement), lifestyle risk factors (fruit and vegetable intake, physical activity, smoking) and self-reported medically diagnosed chronic diseases (diabetes mellitus, hypertension, and hypercholesterolemia).

Adequate consumption of fruits and vegetables was based on the Malaysian Dietary Guidelines (MDG) 2010 [23], which recommended daily consumption of at least two servings of fruits and three servings of vegetables. Physical activity was measured using the validated Global Physical Activity Questionnaire (GPAQ) [24]. Participants with a total physical activity level of at least 600 metabolic equivalent task (MET) minutes per week were considered as being physically active [25]. Current smoking was defined as currently using any smoked tobacco products (manufactured cigarettes, hand-rolled cigarettes, kretek, cigars, shisha, bidis, or tobacco pipes) at the time of the survey.

The self-reported information on the presence of medically diagnosed diabetes, hypertension, and hypercholesterolemia was based on a series of questions: “Have you ever been told by a doctor or Assistant Medical Officer that you have high blood sugar/diabetes, high blood pressure/hypertensive or that you have high blood cholesterol/hypercholesterolemia?”, respectively. Participants who answered “yes” to the questions were considered as having medically diagnosed diabetes, hypertension or hypercholesterolemia.

Bodyweight and height were measured by trained data collectors using TANITA Digital Weighing Scale HD 319 (TANITA Corp., Tokyo, Japan) and SECA Portable Stadiometer 213 (SECA GmbH and Co. KG, Hamburg, Germany), respectively. Body mass index (BMI) was calculated as bodyweight divided by height squared (kg/m^2^). BMI was classified based on the WHO’s criteria: underweight (<18.5), normal (18.5–24.9), overweight (25.0–29.9), and obese (≥30) [26]. Participants with a BMI ≥ 25 kg/m^2^ were classified as overweight/obese.

### 2.3. Clustering of Modifiable CVD Risk Factors

There were five modifiable CVD risk factors included in the current study: (1) Self-reported medically diagnosed diabetes, (2) self-reported medically diagnosed hypertension, (3) self-reported medically diagnosed hypercholesterolemia, (4) overweight/obesity (BMI ≥ 25 kg/m^2^), and (5) current smoking. Each respondent was given a score of 1 each for the presence of self-reported diabetes, hypertension, hypercholesterolemia, overweight/obesity, and current smoking. If the respondent had no risk factor, the score was “0”. For analyses in this study, modifiable CVD risk factor clustering was grouped into four categories: 0 (no risk factor), ≥1, ≥2, and ≥3 CVD risk factors. The modifiable CVD risk factor clusters of ≥1, ≥2, and ≥3 risk factors versus no risk factor (reference group) were analyzed through a logistic regression model.

### 2.4. Statistical Analysis

Descriptive statistics were performed to describe the sociodemographic characteristics of the study population. The prevalence of each CVD risk factor and CVD risk factor clustering (0, ≥1, ≥2, and ≥3) were described by sex (male, female), age group (50–59 years, 60–69 years, 70–79 years, ≥80 years), ethnicity (Malays, Chinese, Indians, Bumiputera Sabah, Bumiputera Sarawak, Others), residential area (urban, rural), marital status (married, unmarried/separated/divorced/widowed), education level (no formal education, primary, secondary, tertiary), employment status (employed, unemployed/retiree/homemaker), individual monthly income (<MYR1000, MYR1000-MYR1999, ≥MYR2000), living arrangement (living alone, living with someone), consumption of fruits (<2 servings/day, ≥2 servings/day), consumption of vegetables (<3 servings/day, ≥3 servings/day), and physical activity status (active, inactive). Differences in prevalence values for categorical variables were compared using χ^2^ test. Univariable logistic regression was first applied to examine the association between each independent variable and the clustering of CVD risk factors (≥1, ≥2, and ≥3 risk factors versus “0” risk factor). Then, multivariable logistic regression analysis was performed to explore the association of relevant characteristics with ≥1, ≥2, and ≥3 CVD risk factor clustering. For the multivariable logistic regression, the multicollinearity and interaction problems were checked. The goodness of fit of the logistic regression model was assessed using Nagelkerke pseudo R-squared (pseudo-*R*^2^). The adjusted odds ratios (ORs) and 95% confidence intervals (CIs) were presented. A *p*-value of <0.05 was considered statistically significant. All statistical analyses were performed using the Statistical Package of Social Sciences (SPSS) for Windows version 25.0 (IBM, Armonk, NY, USA), considering the sample weightage and complex sampling design.

## 3. Results

Table 1 shows the characteristics of the study participants. Of the total 7117 participants, the numbers of males (49.7%) and females (50.3%) were almost equal, with an overall mean age of 62.2 ± 8.8 years. Nearly half of the participants were of age group 50–59 years (48.5%), followed by age groups 60–69 years (34.3%), 70–79 years (13.4%), and ≥80 years (3.9%). Almost three-fifths (56.6%) of the participants were Malays, the major ethnic group in the Malaysian population. The majority of the participants were urban dwellers (75.1%), married (76.5%), and living with someone (95.8%). Only a small percentage of participants (9.7%) did not have formal education, 33.8% had primary education, 43.6% had secondary education, and 12.9% had tertiary education. The majority were unemployed/retirees/homemakers (58.0%). Close to half of the participants (48.4%) had an individual monthly income of less than MYR1000. Nearly 90% of the participants did not consume adequate servings of fruits (88.4%) and vegetables (88.9%), and 23.4% of the participants were physically inactive.

As shown in Table 2, the prevalence of modifiable CVD risk factors, including self-reported diabetes, hypertension, hypercholesterolemia, overweight/obesity, and current smoking, was 23.3%, 42.2%, 35.6%, 58.4%, and 17.5%, respectively. The prevalence of self-reported hypertension, hypercholesterolemia, and overweight/obesity was significantly higher in females than in males (all *p* < 0.001), except for current smoking, where a significantly higher prevalence was observed in males than in females. The prevalence of self-reported diabetes did not differ significantly by sex (*p* > 0.05). Among the older adult population, the peak prevalence of diabetes, hypertension, and hypercholesterolemia appeared in the 70–79-year-old age group, while the prevalence of overweight/obesity and smoking was highest among the 50–59-year-old adults and progressively reduced with age. There were significant differences in the prevalence of CVD risk factors among different ethnic groups (*p* < 0.05). The prevalence of self-reported diabetes was highest in the Indians (35.6%). Bumiputera Sarawak ethnic group had the highest prevalence of self-reported hypertension (60.7%) and hypercholesterolemia (40.7%). The prevalence of overweight/obesity and current smoking was highest in the Malays (63.7%) and Others (20.6%), respectively. No significant difference in the prevalence of CVD risk factors was observed across urban-rural residential areas, except for current smoking, which showed a significantly higher prevalence in rural areas than in urban areas (*p* < 0.001). The prevalence of self-reported hypertension was significantly higher among older adults who were unmarried/separated/divorced/widowed (48.1%) than their married counterparts (*p* < 0.001). Married individuals reported a significantly higher prevalence of current smoking (19.2%) than their unmarried counterparts (*p* < 0.001). The prevalence of self-reported diabetes, hypertension, and hypercholesterolemia were higher in the lower education and lower income groups. The prevalence of overweight/obesity increased with increasing education levels. Older adults with secondary education levels had the highest prevalence of current smoking (19.8%) compared to other levels of education. The prevalence of self-reported diabetes mellitus, hypertension, and hypercholesterolemia were significantly higher among those who were unemployed/retirees/homemakers (all *p* < 0.001). Smoking was more prevalent among older adults who were employed (27.7%) compared to their unemployed counterparts (*p* < 0.001). Older adults who were living with someone had a significantly higher prevalence of overweight/obesity (58.9%) than those living alone (*p* < 0.05). There was a significantly higher prevalence of self-reported diabetes (28.3%) and hypertension (50.9%) in the physically inactive group than the active group (*p* < 0.001). 

Table 3 shows that the prevalence of having 0, ≥1, ≥2, and ≥3 modifiable CVD risk factors among older adults aged ≥50 years was 16.7%, 83.3%, 75.4%, and 62.6%, respectively. The prevalence of those without CVD risk factors was higher in females than in males (18.7% vs. 14.8%). The prevalence of having ≥1, ≥2, and ≥3 modifiable CVD risk factors was higher in males than in females. The peak prevalence of ≥1, ≥2, and ≥3 modifiable CVD risk factors was found in the age group of 60–69 years. The prevalence of ≥1, ≥2, and ≥3 modifiable CVD risk factors differed significantly by ethnicity. Being married and unemployed/retiree/homemaker appeared to have a higher prevalence of having ≥1, ≥2, and ≥3 modifiable CVD risk factors compared to their respective counterparts. The prevalence of ≥1, ≥2, and ≥3 modifiable CVD risk factors did not differ significantly by residential area, education level, living arrangement, consumption of fruits and vegetables, and physical activity status.

Table 4 presents the multivariable logistic regression analysis for the modifiable CVD risk factor clustering. The adjusted OR of having ≥1 or ≥2 modifiable CVD risk factors was significantly higher among males than females. Compared to the 50–59 years age group, the adjusted OR of having ≥2 or ≥3 modifiable CVD risk factors was significantly higher in the 60–69 years age group. Malays, Indians, and Bumiputera Sarawak ethnicities were more likely to have a clustering of ≥1, ≥2, or ≥3 modifiable CVD risk factors compared to the Chinese ethnic group. Older adults who had no formal education were nearly two times more likely to have ≥2 modifiable CVD risk factors compared to the tertiary education group. The likelihood of having ≥1, ≥2, or ≥3 modifiable CVD risk factors was also greater among those who were unemployed/retirees/homemakers compared to employed individuals. Those who were unmarried/separated/divorced/widowed were significantly less likely to have ≥3 modifiable CVD risk factors compared to their married counterparts. The odds of having ≥2 or ≥3 modifiable CVD risk factors were significantly higher among older adults who were physically inactive. There were no significant associations between clustering of CVD risk factors with individual income level, living arrangement, consumption of fruits and vegetables in the adjusted model.

## 4. Discussion

This study reports the prevalence and clustering of reported modifiable CVD risk factors by sociodemographic and lifestyle characteristics among adults aged 50 years and older in Malaysia. The results demonstrate that the prevalence of CVD risk factors was fairly high, and over 80% of participants had at least one CVD risk factor. Three-quarters of the population had two or more CVD risk factors, and three out of five older adults had three risk factors or more, indicating an increased risk for CVD morbidity and mortality among the older population.

Compared to the previous study on clustering of CVD risk factors among the Malaysian adult population aged ≥18 years [17], the higher prevalence of self-reported diabetes, hypertension, hypercholesterolemia, and overweight/obesity in this study may be because the study sample is of older age group (≥50 years), and the prevalence of most CVD risk factors increase with age [18]. The prevalence of current smoking among older adults in this study was lower than that among Malaysian adults aged ≥18 years [17], as smoking was more prevalent among younger persons in their 30′s and 40′s [18]. Women had a higher prevalence of hypertension, hypercholesterolemia, and overweight/obesity, while smoking prevalence was much higher among men than women (33.7% vs. 1.4%). The higher prevalence of smoking among men in Malaysia was in concordance with previous studies [14,17,18], and this may be explained by the influence of cultural norms and positive beliefs about the social acceptability of smoking that increases the susceptibility of smoking among men [27].

Our findings showed that a high proportion of the older population in Malaysia presented a clustering of ≥1, ≥2, or ≥3 CVD risk factors. According to data from the 2006 NHMS in Malaysia, Selvarajah et al. reported that 63% of the participants had ≥1 CVD risk factor, 33% had ≥2, and 14% had ≥3 out of four biological risk factors (hypertension, hyperglycemia, hypercholesterolemia, and central obesity) for adults aged 18 years and above [17]. Data from the Malaysia Non-Communicable Disease Surveillance-1 (MyNCDS-1) 2005/2006 showed that the prevalence of having ≥3 CVD risk factors was 68.4% for 24–64 year-old adults [14]. When compared with findings from the neighboring country on clustering of five CVD biological risk factors (overweight/obesity, central obesity, hypertension, dyslipidemia, and diabetes) among older adults aged ≥65 years in Shenzhen City, China, the prevalence of having at least one CVD risk factor in our study (83.3%) was almost comparable with that reported in China (86.0%) [28]. However, in our study, the prevalence of having ≥2 (75.4%) and ≥3 CVD risk factors (62.6%) was worse than those reported in the Chinese study (≥2 risk factors, 60.8%; ≥3 risk factors, 33.8%) [28]. The differences in the prevalence of clustering of CVD risk factors across different studies could be attributed to differences in the studied population, age range, definitions, and diagnostic criteria used in those studies [28].

In this study, men were more likely to present clustering of modifiable CVD risk factors compared to women. The gender discrepancy in modifiable CVD risk factor clustering was also observed in several other studies [17,18,28,29], depending on the types of CVD risk factor investigated in those studies. We speculated that the inclusion of smoking as one of the CVD risk factors in this study may be somehow increased the tendency of risk factor clustering in men, as smoking was more common among men than women. In addition, it was found that the clustering of risk factors increased with increasing age up to 60–69-year-old group, but decreased thereafter. One of the possible reasons for this observation is that the competing risk for non-CVD morbidities, such as chronic lung disease and cancer that occurs after 70 years of age [30]. With regards to ethnicity, our study showed that Malays, Indians, and Bumiputera Sarawak ethnic groups were more likely to have ≥1, ≥2, or ≥3 risk factors compared to Chinese. Older adults of Bumiputera Sarawak ethnicity had the highest odds of modifiable CVD risk factor clustering in this study, suggesting they are at the highest risk of developing CVD relative to the other ethnicities. The ethnic differences in modifiable CVD risk factor clustering may be due to differences in cultural socioeconomic circumstances, behavioral and dietary factors, as well as genetic factors that deserved further research [14,31].

The prevalence of clustering of modifiable CVD risk factors seems to be almost similar between older populations in urban and rural areas. In the multivariable analysis, after adjusting for all other covariates, the results suggested that urban older adults were associated with a higher likelihood of having ≥1 and ≥2 modifiable CVD risk factors compared to their rural counterparts. This may be explained by the rapid urbanization and consequential changes in lifestyle and diet that are experienced by urban residents, which has probably contributed to the higher prevalence of CVD risk factor clustering, in accordance with results from other studies [31,32]. Considering marital status, we found older adults who were unmarried/separated/divorced/widowed were less likely to have three or more modifiable CVD risk factors compared to their married counterparts, but no significant association was observed between marital status and clustering of ≥1 and ≥2 risk factors. A recently published meta-analysis indicated that inconsistent results have been documented on the relationship between marital status and CVD risk in men and women, and the association between marital status and CVD risk varied by gender [33]. Another possible explanation for the inconsistent association between marital status and CVD risk factor clustering in our study may be that other measures of socioeconomic and behavioral risk factors contribute to the development of modifiable CVD risk factor clustering in our population.

Previous studies showed that CVD risk factor clustering was negatively associated with the level of socioeconomic status (SES), which is the measure of education level, income, and occupation [14,29,34]. A lower SES is usually correlated with poor health and vice versa. Our study revealed that clustering of modifiable CVD risk factors was negatively associated with education level. However, significant results were only observed in the adjusted model for ≥2 risk factors, in which older adults with no formal education had 1.7 times higher odds of developing ≥2 risk factors compared to those with tertiary education level. In terms of employment status, we found that being unemployed, retirees and homemakers were more likely to have modifiable CVD risk factor clustering compared to those being employed, which is consistent with findings reported by Hong et al. [29]. A recently published review article showed that four markers for SES—low income, low educational attainment, unemployment, and disadvantaged neighborhood characteristics (e.g., absence of sidewalks or recreational spaces, unavailability of affordable healthy food, lack of neighborhood safety, and lack of community cohesion) were socioeconomic factors leading to increased CVD risk [35]. However, environmental factors, such as neighborhood socioeconomic characteristics, were not investigated in our study. Longitudinal studies suggested that retirement has different effects on health depending on the health outcomes studied, the context of the country, and the study population [36]. The positive association between retired people and CVD risk factor clustering in our study may be linked to their old age which increased their risks for cardiovascular diseases. Previous local studies showed that being a housewife increased the risk of developing multiple cardiovascular risk factors, particularly low-income housewives who were more likely to be physically inactive, overweight, or obese, which puts them at-risk of cardiovascular disease [14,37]. This may explain the higher likelihood of having CVD risk factor clustering among homemakers who are mostly housewives in our study.

Unhealthy lifestyle behaviors, such as high alcohol consumption, low fruit and vegetable intake, physical inactivity, and sedentary behavior, are known to increase CVD risk [38]. In this study, older adults who were physically inactive were more likely to present ≥2 or ≥3 modifiable CVD risk factors, which was consistent with other reports [29,39]. Shi et al. identified an inverse dose-response association between physical activity and clustering of CVD modifiable risk factors among Chinese adults, suggesting about 15% decreased prevalence of having ≥2 CVD risk factors might be avoided if the inactive individuals (<600 MET-min/week) improved their physical activity to moderate level (600–3000 MET-min/week) [39]. Although the intensity, mode, duration, and frequency of physical activity can strongly affect the outcome, older adults who often have multiple health conditions are encouraged to be as physically active as possible to gain more health benefits from physical activity. Potential biological mechanism underlying the cardiovascular protective effects of physical activity against the clustering of CVD modifiable risk factors include some favorable modification effects in adiposity, insulin sensitivity, lipid profiles, and systemic inflammation [40].

Although a high fruit and vegetable intake has been found to be associated with reduced risk of cardiovascular disease and all-cause mortality [41], no significant association was observed between fruit and vegetable consumption and modifiable CVD risk factors clustering in our study. This may be since fruit and vegetable consumption pattern is still very low among Malaysian adults, with over 90% of the Malaysian adult population did not consume adequate fruits and/or vegetables as recommended by WHO and MDG [12]. The low intake of fruits and vegetables among older adults in this study and the possibility of self-reporting bias may have hampered the association between fruit and vegetable consumption and modifiable CVD risk factors clustering. Additionally, the types and amounts of fruits and vegetables consumed may react differently in reducing CVD risk [41], since our study did not capture types of fruit and vegetable consumed.

The major strength of this study is the stratified cluster sampling design to ensure a nationally representative sample which enables the findings to be generalizable to the older population aged 50 years and above in Malaysia. The large sample size ensures adequate statistical power when estimating the prevalence and clustering of reported modifiable CVD risk factors. Additionally, a high response rate, use of standardized questionnaires or tools, face-to-face data collection by trained interviewers, and good quality control throughout the survey period ensured the validity of our self-reported data. This study also has several limitations worth mentioning. First, the cross-sectional design of the survey prevents determining the causal-effect relationship between modifiable CVD risk factors clustering and the incidence of CVD. Second, the information on modifiable CVD risk factors, such as diabetes, hypertension, and hypercholesterolemia, were assessed based on self-report and did not involve clinical measurements at the time of the survey. However, self-report measures of diabetes, hypertension, and hypercholesterolemia are common in epidemiological surveys, as objective measurements are costly and not economically feasible. In addition, the accuracy of the self-reported data was highly correlated with physicians’ records, as mentioned in previous studies [42,43,44]. Self-reported information on smoking, physical activity, and fruit and vegetable intake may also be subjected to social desirability bias, recall bias, and over- or under-reporting. Response bias appeared to be an unavoidable problem in epidemiological survey research. However, the use of appropriate study design, data collection by trained interviewers, and careful monitoring of data collection procedures by field supervisors who are health care staff could help to minimize the response bias and assure the quality of the responses in this study. Objective measurement of physical activity (e.g., pedometer or accelerometer) and clinical assessment of cardiovascular risk should be considered in future studies to improve data accuracy.

## 5. Conclusions

Our findings indicate that men, age group 60–69 years, urban dwellers, those with no formal education, unemployed/retirees/homemakers, and those being physically inactive are vulnerable subgroups susceptible to modifiable CVD risk factors clustering. This study contributes to the scarce literature on the clustering of reported modifiable CVD risk factors and their association with sociodemographic and lifestyle characteristics among older adults in Malaysia. The findings provide evidence that targeted public health programs are required to enhance public health awareness, strengthen health promotion programs, and improve the health of the older population. Early detection, prevention, and intervention strategies are crucial to reduce the prevalence and clustering of modifiable CVD risk factors. Additionally, findings from this study support the clinical importance of assessing multiple risk factors rather than traditional approaches that target a single risk factor for CVD prevention and management. The current study provides an important public health message and reminds health professionals to assess related risk factors when one risk factor is detected. Future research should focus on identifying possible mechanisms or pathways underlying the clustering of CVD risk factors. A prospective cohort study should be considered in the future to evaluate the impact of clustering and predict modifiable CVD risk factors, including biomarker profiles for CVD risk prediction.

## Figures and Tables

**Table 1:** Descriptive characteristics of the study participants (*n* = 7117), NHMS 2018.

Variables	Unweighted Count (*n*)	Percentage (%)
Sex		
Male	3327	49.7
Female	3790	50.3
Age group (years)		
50–59	3140	48.5
60–69	2563	34.3
70–79	1104	13.4
≥80	310	3.9
Ethnicity		
Malays	4555	56.6
Chinese	1143	24.9
Indians	261	7.7
Bumiputera Sabah	594	4.4
Bumiputera Sarawak	303	3.9
Others	261	2.4
Residential area		
Urban	3102	75.1
Rural	4015	24.9
Marital status		
Married	5269	76.5
Unmarried/separated/divorced/widowed	1844	23.5
Education level		
No formal education	1035	9.7
Primary	2797	33.8
Secondary	2618	43.6
Tertiary	667	12.9
Employment status		
Employed	2897	42.0
Unemployed/retiree/homemaker	4220	58.0
Individual monthly income (MYR) †		
<MYR1000	3953	48.4
MYR1000-MYR1999	1645	22.8
≥MYR2000	1442	28.8
Living arrangement		
Living alone	362	4.2
Living with someone	6755	95.8
Consumption of fruits		
<2 servings/day	6334	88.4
≥2 servings/day	783	11.6
Consumption of vegetables		
<3 servings/day	6272	88.9
≥3 servings/day	814	11.1
Physical activity status		
Active	5270	76.6
Inactive	1838	23.4

† MYR1.00 = USD 0.24 on 16 December 2019.

**Table 2:** The prevalence (95% CI) of modifiable risk factors of cardiovascular disease (self-reported diabetes, hypertension, hypercholesterolemia, overweight/obesity, and current smoking) by sociodemographic and lifestyle characteristics among older adults.

Variables	Self-Reported Diabetes	Self-Reported Hypertension	Self-Reported Hypercholesterolemia	Overweight/Obesity	Current Smoking
Overall	23.3 (21.8 to 25.0)	42.2 (40.3 to 44.1)	35.6 (33.8 to 37.5)	58.4 (56.3 to 60.5)	17.5 (16.0 to 19.0)
Sex					
Male	22.2 (20.3 to 24.2)	38.2 (35.8 to 40.6)	32.3 (30.1 to 34.6)	53.4 (50.8 to 55.9)	33.7 (31.1 to 36.4)
Female	24.5 (22.3 to 26.7)	46.2 (43.6 to 48.8)	38.9 (36.3 to 41.5)	63.4 (60.7 to 66.1)	1.4 (1.0 to 1.8)
*p*-value	0.093	<0.001	<0.001	<0.001	<0.001
Age group (years)					
50–59	18.8 (16.7 to 21.0)	32.7 (33.7 to 41.8)	29.1 (26.5 to 31.8)	62.2 (58.8 to 65.4)	21.8 (19.5 to 24.3)
60–69	28.3 (25.4 to 31.5)	48.7 (46.3 to 51.2)	41.8 (39.0 to 44.6)	59.5 (56.5 to 62.5)	14.3 (12.4 to 16.5)
70–79	29.1 (25.4 to 33.2)	57.5 (53.7 to 61.2)	45.5 (41.6 to 49.4)	46.9 (42.7 to 51.1)	11.7 (9.3 to 14.5)
≥80	16.6 (11.6 to 23.2)	49.8 (41.0 to 58.7)	29.6 (22.7 to 37.5)	30.4 (23.1 to 38.9)	10.7 (6.8 to 16.5)
*p*-value	<0.001	<0.001	<0.001	<0.001	<0.001
Ethnicity					
Malays	25.8 (24.1 to 27.6)	41.5 (39.3 to 43.8)	37.1 (35.1 to 39.1)	63.7 (60.9 to 66.3)	20.2 (18.4 to 22.2)
Chinese	17.2 (14.5 to 20.3)	41.5 (37.0 to 46.1)	32.2 (28.3 to 36.3)	46.9 (43.4 to 50.5)	12.8 (10.3 to 15.7)
Indians	35.6 (30.6 to 40.9)	41.7 (35.0 to 48.8)	39.0 (30.8 to 48.0)	62.2 (55.1 to 68.9)	9.9 (6.2 to 15.4)
Bumiputera Sabah	12.7 (9.3 to 17.1)	41.5 (34.2 to 49.2)	30.5 (26.5 to 34.9)	56.6 (49.6 to 63.3)	19.8 (14.6 to 26.1)
Bumiputera Sarawak	21.7 (16.9 to 27.4)	60.7 (51.6 to 69.1)	40.7 (33.5 to 48.2)	52.9 (46.7 to 59.1)	17.7 (14.1 to 22.0)
Others	12.7 (7.9 to 19.7)	36.6 (26.6 to 47.9)	26.7 (18.5 to 36.8)	50.9 (43.9 to 57.9)	20.6 (14.8 to 27.9)
*p*-value	<0.001	0.007	0.038	<0.001	<0.001
Residential area					
Urban	24.0 (22.0 to 26.1)	41.4 (39.1 to 43.9)	36.1 (33.7 to 38.5)	59.2 (56.5 to 61.9)	16.1 (14.3 to 18.0)
Rural	21.5 (19.7 to 23.4)	44.4 (41.7 to 47.1)	34.2 (32.1 to 36.4)	56.0 (53.8 to 58.1)	21.7 (20.1 to 23.4)
*p*-value	0.078	0.105	0.259	0.068	<0.001
Marital status					
Married	23.5 (21.7 to 25.3)	40.3 (38.2 to 42.5)	35.6 (33.7 to 37.5)	59.6 (57.3 to 61.9)	19.2 (17.5 to 21.0)
Unmarried/separated/divorced/widowed	22.9 (20.2 to 25.9)	48.1 (44.7 to 51.4)	35.7 (32.0 to 39.5)	54.1 (50.7 to 57.5)	11.9 (9.9 to 14.1)
*p*-value	0.739	<0.001	0.955	0.073	<0.001
Education level					
No formal education	26.2 (23.0 to 29.8)	54.9 (50.9 to 58.8)	36.9 (32.8 to 41.3)	50.8 (46.5 to 55.0)	12.0 (9.4 to 15.2)
Primary	24.5 (22.2 to 26.9)	45.2 (42.6 to 47.9)	37.4 (34.7 to 40.1)	55.4 (52.4 to 58.2)	18.0 (16.2 to 20.0)
Secondary	22.0 (19.7 to 24.5)	39.5 (36.6 to 42.4)	35.5 (32.9 to 38.2)	60.0 (56.8 to 63.0)	19.8 (17.6 to 22.2)
Tertiary	22.7 (18.9 to 27.0)	33.8 (28.8 to 39.2)	30.4 (25.8 to 35.4)	65.9 (61.5 to 70.1)	12.1 (9.1 to 15.8)
*p*-value	0.192	<0.001	0.046	<0.001	<0.001
Employment status					
Employed	17.1 (15.2 to 19.1)	32.0 (29.5 to 34.7)	28.4 (26.0 to 30.8)	56.3 (53.5 to 59.1)	27.7 (25.1 to 30.4)
Unemployed/retiree/homemaker	27.9 (25.7 to 30.1)	49.5 (47.3 to 51.8)	40.9 (38.5 to 43.3)	60.0 (57.5 to 62.3)	10.0 (8.8 to 11.4)
*p*-value	<0.001	<0.001	<0.001	0.125	<0.001
Individual monthly income (MYR)					
<MYR1000	25.0 (23.0 to 27.1)	46.7 (44.3 to 49.2)	37.9 (35.3 to 40.4)	55.8 (53.2 to 58.3)	12.6 (11.1 to 14.2)
MYR1000-MYR1999	23.1 (20.2 to 26.3)	40.9 (37.1 to 44.8)	35.2 (31.8 to 38.7)	61.7 (57.7 to 65.5)	24.7 (21.7 to 27.9)
≥MYR2000	21.1 (18.6 to 23.8)	35.5 (32.2 to 38.9)	32.3 (29.3 to 35.5)	60.4 (57.1 to 63.6)	20.5 (17.7 to 23.6)
*p*-value	0.049	<0.001	0.016	0.081	<0.001
Living arrangement					
Living alone	25.7 (18.5 to 34.4)	47.6 (40.4 to 54.9)	34.6 (27.4 to 42.7)	47.9 (39.5 to 56.4)	21.5 (16.0 to 28.2)
Living with someone	23.2 (21.7 to 24.8)	41.9 (40.0 to 43.9)	35.7 (33.9 to 37.5)	58.9 (56.7 to 61.0)	17.3 (15.8 to 18.8)
*p*-value	0.519	0.122	0.782	0.013	0.146
Consumption of fruits					
<2 servings/day	23.4 (21.9 to 25.1)	42.7 (40.5 to 44.9)	35.7 (33.6 to 37.7)	57.8 (55.6 to 59.9)	17.8 (16.4 to 19.4)
≥2 servings/day	22.8 (18.6 to 27.6)	38.1 (34.1 to 42.4)	35.4 (31.1 to 40.0)	63.0 (58.2 to 67.5)	14.5 (11.5 to 18.3)
*p*-value	0.791	0.073	0.931	0.059	0.085
Consumption of vegetables					
<3 servings/day	23.5 (21.8 to 25.2)	42.3 (40.3 to 44.4)	36.0 (34.0 to 38.1)	58.1 (55.8 to 60.4)	17.5 (16.1 to 19.1)
≥3 servings/day	22.8 (19.0 to 27.2)	41.1 (36.3 to 46.0)	33.0 (29.0 to 37.3)	60.5 (55.7 to 65.1)	17.1 (13.7 to 21.2)
*p*-value	0.780	0.635	0.210	0.380	0.835
Physical activity status					
Active	21.8 (20.2 to 23.6)	39.5 (37.5 to 41.6)	34.6 (32.6 to 36.8)	59.3 (56.9 to 61.6)	17.1 (15.5 to 18.8)
Inactive	28.3 (25.2 to 31.6)	50.9 (47.0 to 54.8)	38.9 (35.8 to 42.1)	55.0 (51.6 to 58.4)	18.6 (16.1 to 21.4)
*p*-value	<0.001	<0.001	0.175	0.124	0.302

**Table 3:** The prevalence (95% CI) with different numbers of modifiable cardiovascular disease (CVD) risk factors.

Variables	Number of Modifiable CVD Risk Factors Clusters			
	None (0) (*n* = 481)	≥1 (*n* = 2846)	≥2 (*n* = 1710)	≥3 (*n* = 880)
Overall	16.7 (15.2 to 18.54)	83.3 (81.6 to 84.8)	75.4 (72.9 to 77.7)	62.6 (59.2 to 65.9)
Sex				
Male	14.8 (13.1 to 16.5)	85.2 (83.5 to 86.9)	78.1 (75.4 to 80.5)	64.8 (61.6 to 68.4)
Female	18.7 (16.5 to 21.1)	81.3 (78.9 to 83.5)	72.8 (69.4 to 75.9)	60.7 (56.3 to 64.9)
*p*-value	-	0.002	0.001	0.069
Age group (years)				
50–59	16.6 (14.0 to 19.6)	83.4 (80.4 to 86.0)	73.3 (68.5 to 77.5)	59.2 (52.9 to 65.2)
60–69	14.9 (12.9 to 17.2)	85.1 (82.8 to 87.1)	79.5 (76.2 to 82.5)	69.1 (64.4 to 73.4)
70–79	16.7 (14.6 to 19.0)	83.3 (81.0 to 85.4)	77.5 (74.3 to 80.5)	65.9 (61.4 to 70.2)
≥80	34.4 (26.0 to 44.0)	65.6 (56.0 to 74.0)	54.8 (44.2 to 64.9)	31.2 (20.6 to 44.3)
*p*-value	-	<0.001	<0.001	<0.001
Ethnicity				
Malays	13.4 (12.0 to 14.9)	86.6 (85.1 to 88.0)	80.3 (78.0 to 82.5)	69.6 (66.3 to 72.7)
Chinese	24.7 (21.5 to 28.2)	75.3 (71.8 to 78.5)	64.0 (58.7 to 69.0)	46.2 (39.9 to 52.6)
Indians	14.6 (9.5 to 21.8)	85.4 (78.2 to 90.5)	78.4 (69.0 to 85.6)	69.5 (57.1 to 79.6)
Bumiputera Sabah	16.8 (13.8 to 20.3)	83.2 (79.7 to 86.2)	73.7 (67.4 to 79.1)	55.7 (46.7 to 64.3)
Bumiputera Sarawak	14.1 (10.8 to 18.2)	85.9 (81.8 to 89.2)	79.9 (73.9 to 84.8)	71.8 (63.5 to 78.8)
Others	24.4 (17.5 to 32.9)	75.6 (67.1 to 82.5)	62.6 (51.4 to 72.6)	45.6 (30.6 to 61.5)
*p*-value	-	<0.001	<0.001	<0.001
Residential area				
Urban	16.9 (14.9 to 19.1)	83.1 (80.9 to 85.1)	75.3 (72.1 to 78.4)	62.4 (58.0 to 66.6)
Rural	16.4 (15.2 to 17.7)	83.6 (82.3 to 84.8)	75.5 (73.6 to 77.3)	63.1 (60.4 to 65.8)
*p*-value	-	0.699	0.951	0.788
Marital status				
Married	15.9 (14.2 to 17.8)	84.1 (82.2 o 85.8)	76.3 (73.6 to 78.8)	64.3 (60.6 to 67.9)
Unmarried/separated/divorced/widowed	19.4 (16.8 to 22.2)	80.6 (77.8 to 83.2)	72.4 (68.5 to 76.1)	56.9 (52.1 to 61.5)
*p*-value	-	0.020	0.050	0.004
Education level				
No formal education	18.8 (16.1 to 21.9)	81.2 (78.1 to 83.9)	73.9 (69.8 to 77.6)	60.3 (54.9 to 65.4)
Primary	16.8 (15.0 to 18.8)	83.2 (81.2 to 85.0)	75.5 (72.6 to 78.2)	63.2 (59.4 to 66.7)
Secondary	15.8 (13.6 to 18.4)	84.2 (81.6 to 86.4)	76.6 (72.8 to 80.0)	64.0 (58.8 to 69.0)
Tertiary	18.0 (14.6 to 22.0)	82.0 (78.0 to 85.4)	71.9 (65.8 to 77.4)	58.1 (50.4 to 65.5)
*p*-value	-	0.359	0.329	0.370
Employment status				
Employed	16.9 (14.9 to 19.2)	83.1 (80.8 to 85.1)	73.2 (69.5 to 76.5)	56.2 (51.1 to 61.2)
Unemployed/retiree/homemaker	16.6 (14.9 to 18.5)	83.4 (81.5 to 85.1)	76.8 (74.1 to 79.3)	66.3 (62.8 to 69.6)
*p*-value	-	0.791	0.042	<0.001
Individual monthly income (MYR)				
<MYR1000	18.2 (16.3 to 20.4)	81.8 (79.6 to 83.7)	73.8 (70.8 to 76.6)	61.5 (57.7 to 65.2)
MYR1000-MYR1999	13.0 (10.9 to 15.5)	87.0 (84.5 to 89.1)	80.6 (76.9 to 83.9)	69.0 (63.3 to 74.1)
≥MYR2000	16.7 (14.3 to 19.3)	83.3 (80.7 to 85.7)	74.6 (70.6 to 78.3)	60.7 (55.3 to 65.9)
*p*-value	-	0.002	0.005	0.027
Living arrangement				
Living alone	17.3 (12.8 to 22.9)	82.7 (77.1 to 87.2)	74.8 (66.9 to 81.3)	63.0 (51.6 to 73.1)
Living with someone	16.7 (15.1 to 18.4)	83.3 (81.6 to 84.9)	75.4 (72.9 to 77.8)	62.6 (59.2 to 65.9)
*p*-value	-	0.825	0.863	0.947
Consumption of fruits				
<2 servings/day	16.8 (15.2 to 18.6)	83.2 (81.4 to 84.8)	75.3 (72.7 to 77.7)	62.7 (59.2 to 66.0)
≥2 servings/day	16.0 (12.8 to 19.8)	84.0 (80.2 to 87.2)	76.1 (70.7 to 80.7)	62.2 (54.7 to 69.2)
*p*-value	-	0.635	0.756	0.906
Consumption of vegetables				
<3 servings/day	16.8 (15.1 to 18.6)	83.2 (81.4 to 84.9)	75.3 (72.6 to 77.9)	63.0 (59.3 to 66.5)
≥3 servings/day	15.5 (12.0 to 19.8)	84.5 (80.2 to 88.0)	77.3 (71.3 to 82.3)	61.5 (52.8 to 69.6)
*p*-value	-	0.557	0.539	0.752
Physical activity status				
Active	16.8 (15.0 to 18.8)	83.2 (81.2 to 85.0)	74.9 (72.0 to 77.5)	61.7 (57.7 to 65.5)
Inactive	16.3 (14.1 to 18.7)	83.7 (81.3 to 85.9)	77.2 (73.8 to 80.4)	65.8 (61.2 to 70.1)
*p*-value	-	0.720	0.222	0.142

**Table 4:** Adjusted odds ratio (OR) and 95% confidence interval (CI) of the modifiable cardiovascular disease risk factor clustering among older adults.

Variables	≥1 Risk Factors		≥2 Risk Factors		≥3 Risk Factors	
	Adjusted OR (95% CI)	*p*-Value	Adjusted OR (95% CI)	*p*-Value	Adjusted OR (95% CI)	*p*-Value
Sex						
Male	1.37 (1.13 to 1.67)	0.002	1.40 (1.14 to 1.73)	0.002	1.26 (0.99 to 1.60)	0.059
Female	1.00		1.00		1.00	
Age group (years)						
50–59	1.00		1.00		1.00	
60–69	1.10 (0.86 to 1.41)	0.455	1.38 (1.05 to 1.83)	0.023	1.46 (1.08 to 1.98)	0.015
70–79	0.95 (0.71 to 1.27)	0.704	1.18 (0.85 to 1.62)	0.327	1.20 (0.84 to 1.70)	0.324
≥80	0.32 (0.19 to 0.55)	<0.001	0.37 (0.21 to 0.65)	0.001	0.23 (0.12 to 0.45)	<0.001
Ethnicity						
Malays	2.21 (1.74 to 2.82)	<0.001	2.38 (1.80 to 3.16)	<0.001	2.84 (2.07 to 3.88)	<0.001
Chinese	1.00		1.00		1.00	
Indians	2.01 (1.27 to 3.18)	0.003	2.19 (1.37 to 3.51)	0.001	3.07 (1.84 to 5.11)	<0.001
Bumiputera Sabah	1.76 (1.23 to 2.52)	0.002	1.71 (1.12 to 2.63)	0.014	1.66 (0.98 to 2.83)	0.061
Bumiputera Sarawak	2.34 (1.60 to 3.41)	<0.001	2.64 (1.75 to 3.99)	<0.001	3.73 (2.34 to 5.93)	<0.001
Others	1.07 (0.66 to 1.76)	0.777	1.04 (0.62 to 1.75)	0.873	1.17 (0.59 to 2.33)	0.649
Residential area						
Urban	1.22 (1.01 to 1.48)	0.045	1.27 (1.02 to 1.58)	0.035	1.25 (0.98 to 1.59)	0.067
Rural	1.00		1.00		1.00	
Marital status						
Married	1.00		1.00		1.00	
Unmarried/separated/divorced/widowed	0.91 (0.74 to 1.12)	0.376	0.88 (0.70 to 1.10)	0.248	0.71 (0.55 to 0.91)	0.008
Education level						
No formal education	1.50 (0.98 to 2.28)	0.060	1.70 (1.08 to 2.66)	0.022	1.50 (0.91 to 2.49)	0.112
Primary	1.32 (0.96 to 1.81)	0.085	1.43 (1.00 to 2.04)	0.050	1.46 (0.98 to 2.16)	0.061
Secondary	1.21 (0.91 to 1.63)	0.195	1.35 (0.98 to 1.86)	0.071	1.31 (0.90 to 1.89)	0.158
Tertiary	1.00		1.00		1.00	
Employment status						
Employed	1.00		1.00		1.00	
Unemployed/retiree/homemaker	1.24 (1.02 to 1.52)	0.036	1.42 (1.12 to 1.78)	0.003	1.80 (1.40 to 2.33)	<0.001
Individual monthly income (MYR)						
<MYR1000	0.86 (0.66 to 1.11)	0.250	0.80 (0.60 to 1.06)	0.118	0.73 (0.53 to 1.00)	0.047
MYR1000-MYR1999	1.23 (0.95 to 1.58)	0.120	1.20 (0.90 to 1.61)	0.221	1.08 (0.78 to 1.48)	0.643
≥MYR2000	1.00		1.00		1.00	
Living arrangement						
Living alone	1.10 (0.73 to 1.64)	0.655	1.03 (0.67 to 1.60)	0.891	1.29 (0.75 to 2.22)	0.352
Living with someone	1.00		1.00		1.00	
Consumption of fruits						
<2 servings/day	0.94 (0.72 to 1.24)	0.676	0.97 (0.72 to 1.30)	0.825	0.97 (0.69 to 1.35)	0.853
≥2 servings/day	1.00		1.00		1.00	
Consumption of vegetables						
<3 servings/day	0.87 (0.65 to 1.17)	0.355	0.86 (0.63 to 1.18)	0.353	1.00 (0.69 to 1.44)	0.988
≥3 servings/day	1.00		1.00		1.00	
Physical activity status						
Active	1.00		1.00		1.00	
Inactive	1.17 (0.95 to 1.45)	0.133	1.27 (1.02 to 1.59)	0.035	1.38 (1.07 to 1.77)	0012
Pseudo R2	0.054		0.081		0.134	

Adjusted OR; odds ratio adjusted for all other variables in the model. Multicollinearity and interactions were checked and not found.

## Data Availability

For data protection purposes, the data used for this study are not publicly available but are available from the Institute for Public Health, National Institutes of Health, Ministry of Health Malaysia upon reasonable request and with permission from the Director General of Health Malaysia.

## Abbreviations

BMI	Body mass index
CI	Confidence interval
CVD	Cardiovascular disease
EB	Enumeration block
GPAQ	Global Physical Activity Questionnaire
LMICs	Low- and Middle-Income Countries
LQ	Living quarter
MET	Metabolic equivalent
NHMS	National Health and Morbidity Survey
OR	Odds ratio
WHO	World Health Organization
